# Early *Trypanosoma cruzi* Infection Triggers mTORC1-Mediated Respiration Increase and Mitochondrial Biogenesis in Human Primary Cardiomyocytes

**DOI:** 10.3389/fmicb.2018.01889

**Published:** 2018-08-16

**Authors:** M. Gabriela Libisch, Paula Faral-Tello, Nisha J. Garg, Rafael Radi, Lucía Piacenza, Carlos Robello

**Affiliations:** ^1^Laboratory of Host-Pathogen Interactions-UBM, Institut Pasteur de Montevideo, Montevideo, Uruguay; ^2^Department of Microbiology and Immunology, The University of Texas Medical Branch, Galveston, TX, United States; ^3^Departamento de Bioquímica, Center for Free Radical and Biomedical Research, Facultad de Medicina, Universidad de la República, Montevideo, Uruguay

**Keywords:** Chagas disease, chronic chagasic cardiopathy, host–pathogen, early response to infection, mitochondrial function

## Abstract

Chagasic chronic cardiomyopathy is one of the most frequent and severe manifestations of Chagas disease, caused by the parasite *Trypanosoma cruzi*. The pathogenic and biochemical mechanisms responsible for cardiac lesions remain not completely understood, although it is clear that hypertrophy and subsequent heart dilatation is in part caused by the direct infection of cardiomyocytes. In this work, we evaluated the initial response of human cardiomyocytes to *T. cruzi* infection by transcriptomic profiling. Immediately after infection, cardiomyocytes dramatically change their gene expression patterns, up regulating most of the genes encoding for respiratory chain, oxidative phosphorylation and protein synthesis. We found that these changes correlate with an increase in basal and maximal respiration, as well as in spare respiratory capacity, which is accompanied by mitochondrial biogenesis *pgc-1*α independent. We also demonstrate that these changes are mediated by mTORC1 and reversed by rapamycin, resembling the molecular mechanisms described for the non-chagasic hypertrophic cardiomyopathy. The results of the present work identify that early during infection, the activation of mTORC1, mitochondrial biogenesis and improvement in oxidative phosphorylation are key biochemical changes that provide new insights into the host response to parasite infection and the pathogenesis of chronic chagasic cardiomyopathy. The finding that this phenotype can be reversed opens a new perspective in the treatment of Chagas disease, through the identification of host targets, and the use of combined parasite and host targeted therapies, in order to prevent chagasic cardiomyopathy.

## Introduction

Chagas disease, caused by the protozoan parasite *Trypanosoma cruzi*, remains a major public health problem in Latin America (Bonney, 2014) and more recently has emerged in historically non-endemic regions such as the United States, Canada, Western Europe, Japan, and Australia, due to widespread immigration (Coura and Vinas, 2010; Dumonteil et al., 2012; Bonney, 2014). The parasite penetrates the host through a skin lesion, by contact with mucous tissue, or by ingestion, vertical transmission or transfusion (Coura and Vinas, 2010; Coura, 2015; Meymandi et al., 2017), and is capable of infecting different types of cells, including epithelial and smooth muscle cells, fibroblasts, macrophages, and cardiomyocytes (Fernandes and Andrews, 2012). Clinically, after acute phase that usually resolves spontaneously in 2–4 months, patients remain chronically infected if untreated (Dias et al., 1956; Rassi et al., 2010), and about 30% of them develop chronic chagasic cardiomyopathy (CCC) (Fernandes and Andrews, 2012; Lewis and Kelly, 2016), where the apical aneurysm of the left ventricle constitutes the hallmark of the disease (Matta Guedes et al., 2012; Perez-Molina et al., 2015). At the molecular level, the pathogenesis of CCC remains unclear, since it is a multi-factorial disease where both the direct effect of the parasite and the host immune responses contribute to the progressive cardiac damage. In the initial stages of the infection, the parasite-endothelial interactions are among the first to occur (Tanowitz et al., 2009), and it has been proposed to be responsible for *T. cruzi* induced vasculitis (Morris et al., 1990; Rossi and Bestetti, 1995; Rossi and Ramos, 1996), whereas during the chronic phase, infection of cardiomyocytes becomes relevant, and the disease shows a continuous progression from cardiomyocyte destruction to substitution with fibrosis and compensatory hypertrophy, leading finally to cardiac dilatation (Pereira Barretto et al., 1986; Machado et al., 2012). Simultaneously, the prolonged stimulation of inflammatory responses also contributes to myocarditis, fibrosis, hypertrophic cardiomyopathy (HCM), and then to dilated cardiomyopathy (DCM), with consequent heart failure that can even lead to death (Higuchi et al., 2003).

Transcriptomics studies have shown that in the context of heart disease, CCC has molecular signatures which differentiate it from DCM, such as immune responses, lipid metabolism, and mitochondrial oxidative phosphorylation pathways, selectively up-regulated in myocardial tissue of patients with CCC, when compared with DCM (Cunha-Neto et al., 2005). The expression profiles of myocardial tissues from patients with HCM, a critical step in the progression of CCC, showed that a high number of genes related to protein synthesis and oxidative phosphorylation pathways were up-regulated (Yang et al., 2015), whereas Udoko et al. (2016) found a set of fibrotic-associated genes regulated early during the infection process in human cardiomyocytes (Udoko et al., 2016). *T. cruzi* infection assayed in murine cardiomyocytes (*in vitro* and *in vivo*) showed that hundreds of genes were differentially expressed in response to the infection, most of them related to inflammation processes, immune response, oxidative phosphorylation, and cytoskeleton organization (Garg et al., 2003; Vyatkina et al., 2004; Mukherjee et al., 2008; Goldenberg et al., 2009; Manque et al., 2011). It is noteworthy that most of the gene profiling studies in the context of CCC were performed in murine models and in addition, the initial stages of *T. cruzi* infection in human cardiomyocytes remains poorly understood.

Different works found the activation of AKT in *T. cruzi* infected cells (Chuenkova et al., 2001; Woolsey et al., 2003), and perturbations in the host mTORC1 pathway were also described in *T. cruzi* infected HeLa (Caradonna et al., 2013) and macrophages (Rojas Marquez et al., 2018) cells. The PI3K/AKT pathway, can lead to the phosphorylation of the serine/threonine mTOR kinase which then can form the mammalian target of rapamycin complex 1 (mTORC1). This complex is sensitive to rapamycin treatment and its activation leads to phosphorylation of different substrates including eukaryotic translation initiation factor 4E (eiF4E)-binding proteins (4E-BPs), and ribosomal protein S6 kinases (S6K). The phosphorylation of these substrates can stimulate protein synthesis (Morita et al., 2015). Particularly the phosphorylation of 4E-BP1 has been associated with an increase in mitochondrial activity and biogenesis (Morita et al., 2013).

In this work we analyzed the early response of primary human cardiomyocytes to *T. cruzi* infection, and we found that soon after infection hundred of genes were up-regulated. Pathway analysis revealed the up regulation of genes belonging to mitochondrial energy metabolism and protein synthesis (further confirmed at the metabolic and cell level), via m-TORC1 activation, resembling molecular mechanisms of evolution to cardiac hypertrophy. Together, these results suggest that early *T. cruzi* infection induces metabolic modeling of cardiomyocytes, which may contribute to heart damage in Chagas disease.

## Materials and Methods

### Cell Cultures, Parasites, and Infection Assays

Human primary cardiomyocytes (Celprogen) derived from adult cardiac tissue were grown in Human Cardiomyocyte primary Cell Culture medium (Celprogen) supplemented with 10% (v/v) heat inactivated fetal bovine serum (FBS) (Gibco-BRL) at 37°C in a 5% CO_2_ atmosphere. Dm28c *T. cruzi* strain of TcI lineage was used throughout this work (Contreras et al., 1985). For infection assays, semi-confluent cardiomyocytes were infected with cell-derived trypomastigotes (10:1 parasite: cell ratio), centrifuged for 5 min at 1500 rpm, and incubated for 2 h to allow invasion, at 37°C, 5% CO_2_ in the cardiomyocyte medium without serum. After the interaction period, parasites were removed, and cells were washed 5-times with PBS and incubated with complete cardiomyocyte medium. Cell samples were taken at 0, 3, 6, and 12 h after the interaction period henceforth named *t*_0_, *t*_3_, *t*_6_, and *t*_12_, respectively. In some cases, samples were taken 24 and 48 h post-interaction period. To examine the effect of mTOR inhibition on *T. cruzi* invasion and cellular response, cells were first incubated with 2.5 μM rapamycin (Sigma) for 24 h, washed, and then used for infection assays as described above. Resazurin (Sigma) reduction was used to evaluate the viability of the cells treated with rapamycin, as described by O’Brien et al. (2000).

The Primary Umbilical Vein Endothelial Cells (Huvec) were purchased from ATCC, culture in a specific Vascular Cell Basal Medium (ATCC) supplemented with the Endothelial Cell Growth Kit-BBE (ATCC).

### RNA Extraction and Gene Profiling

Total RNA was isolated with Direct-zol RNA MiniPrep kit (Zymo) as described by the manufacturer, and treated with RNase-free DNase to remove contaminating DNA. Total RNA samples were quantified in a NanoDrop spectrophotometer (NanoDrop Technologies). RNA quality was assessed with Agilent 2100 Bioanalyzer, and only samples with RIN (RNA integrity number) above 7 were used. Microarray analysis was performed by using a Human Gene Expression 8x60K v2 Microarray (Agilent) in a one-color design. Briefly, 200 ng of total RNA was reverse-transcribed into cDNA, and this was transcribed into cRNA and labeled using the Low Input Quick Amp Labeling Kit, One-color (Agilent Technologies). The labeled cRNA was purified with Illustra RNAspin Mini Isolation kit (GE Healthcare, United States). The quality of each cRNA sample was verified by total yield and specificity calculated based on NanoDrop ND-1000 spectrophotometer measurements (NanoDrop Technologies, United States). Then, we proceeded with the hybridization, washing and assembling of the chips according to the protocol specified by Agilent. The slides were scanned using an Agilent microarray scanner G2565BA at default settings for all parameters. We used Agilent Feature Extraction (version 9.5.1) for quality control, data filtering, and data normalization. Three biological replicates were performed for *t*_0_ and *t*_3_ time-points, and two each for *t*_6_ and *t*_12_ time-points. Data processing was performed using GeneSpring GX software package (version 12.0, Agilent Technologies). Genes significantly up- and down-regulated were identified by the ANOVA-test with a *p*-value of 0.01 and a Benjamini–Hochberg false discovery rate correction for multiple testing.

### Quantitative RT-PCR

cDNA was synthesized by reverse transcription using the SuperScript II Reverse Transcriptase (Invitrogen) with Oligo(dT) primers and 500 ng of total RNA added as a template. Primer sequences and expected product length of amplicons are listed in **Supplementary Table S1**. Most of the primers span an exon–exon junction to avoid DNA amplification. Real-time reactions were performed using 5 μL SybrGreen (KAPA SYBR FAST Universal 2X qPCR Master Mix, Kapa Biosystems), 200 nM of forward and reverse primers, and 1 μL of a 1/5th dilution of cDNA, in a final volume of 10 μL. Samples were analyzed in duplicate in an Eco Real-Time PCR System (Illumina). Standard amplification conditions were 3 min at 95°C and 40 cycles of 15 s at 95°C, 30 s at 58°C, and 30 s at 72°C. After each PCR reaction, the corresponding dissociation curves were analyzed to ensure that the desired amplicon was being detected and to discard contaminating DNA or primer dimers. Also agarose gels electrophoresis and Sanger sequencing were done in some cases (*ldhb*, *mdh2*, *rps10*, and *pgc-1*α) to verify the expected PCR product. The threshold cycle (*C*T) value for each gene was normalized to *gapdh*, calculating the Δ*C*t for each gene in all samples 2–3 replicates of control and infected cells at different hours post interaction (hpi). The comparative CT method (ΔΔ*C*t method) was used to determine the relative quantity of the target genes, and the fold change in expression was calculated as 2^-ΔΔ^*^C^^t^*. For measurement of mtDNA, total DNA was extracted with DNAzol kit (Invitrogen). mtDNA levels were measured by a real-time, quantitative PCR using specific primers for the fragment 7122–7285 of the mitochondrial genome RN39 (Sequence ID: MF681706.1). Amplification of mtDNA was measured using the Kapa SYBR fast master mix (Kapa Biosystems), and was expressed relative to the nuclear β –actin gene, using the ΔΔ*C*t method (Livak and Schmittgen, 2001).

### Western Blot and Immunofluorescence Assays

Polyclonal antibody against 4EBP1 (#9452) and monoclonal antibodies against mTORC1 (#2983), phospho-mTOR (#5536), p70S6K (#2708), phospho-p70S6K (#9234), phospho-4EBP1 (#9452), AKT (#4685), phospho-AKT (#2965) phospho-AKT (ser 473) (#4060) were purchased from Cell Signaling Technology. GAPDH antibody was purchased from Sigma. *T. cruzi* protein extracts (20 μg) were loaded and a 12% polyacrylamide gel (SDS-PAGE) were run under reducing conditions and electro-transferred to a nitrocellulose membrane (GE Healthcare). Membranes were blocked in 5% non-fat dry milk in TBS for 16 h at 4°C or 1 h at room temperature. After washing with TBS containing 0.1% Tween 20 (TBST), membranes were incubated with an 1/1000 dilution of antibodies in 5% Bovine Serum Albumin (BSA) (Sigma) in TBST overnight at 4°C, washed and incubated with peroxidase-conjugated goat anti-rabbit or anti-mouse secondary antibody (Sigma). The signal was developed with Super Signal West Pico Chemiluminescent Substrate (Thermo Scientific).

For indirect immunofluorescence (IIF), harvested and washed cells were fixed with 4% paraformaldehyde, and permeabilized and blocked with 0.5% saponin/4% BSA in PBS. Slides were incubated for 1 h with anti-Cyt C antibody (Abcam), diluted 1:100 in 0.5% saponin/4% BSA in PBS. After three washes with PBS-0.5% saponin, slides were incubated with ALEXA-488 conjugated goat anti-mouse IgG (1:1000 dilution, from Invitrogen), and washed 3 times with PBS-0.5% saponin. Finally, slides were mounted with Prolong antifade with DAPI (Invitrogen), and visualized under an Olympus IX 81 microscope coupled to a Hamamatsu Orca-ER camera (Diagnostic Instruments).

### Bioenergetic Profiling of Human Cardiomyocytes

The oxygen consumption rates (QO_2_) and proton production rates (PPR) were measured by using a Seahorse Bioscience XF24 Flux Analyzer (Seahorse Bioscience). For this, cardiomyocytes were seeded (5 × 10^4^ cells/well) and incubated at 37°C in complete media for 24 h before the experiment. The culture media was changed 1 h prior to the assay to unbuffered Dulbecco’s Modified Eagle Medium (DMEM, pH 7.4) supplemented with L-glutamine (2 mM; Gibco), glucose (5 mM) and pyruvate (1 mM). Oligomycin (inhibitor of ATP synthase, complex V), FCCP (uncoupling agent) and a mix of antimycin A (AA, complex III inhibitor) and rotenone (Rot, complex I inhibitor) were injected sequentially through ports in the seahorse flux pack cartridges to final concentrations of 2.0, 1.0, and 1.0 μM, respectively. After subtracting the non-mitochondrial respiration (oxygen consumption in the presence of AA-ROT) from each data, QO_2_ were normalized against cell counts and reported as pmol O_2_/min/10^4^ cells. Basal respiration, maximal respiration (QO_2_ in the presence of FCCP), spare respiratory capacity (QO_2_ with FCCP- basal QO_2_), and the respiratory control ratio (RCR, QO_2_ with FCCP/QO_2_ with oligomycin) were then calculated. Five replicates per condition were analyzed in each plate assay, and at least three independent experiments were performed.

To analyze glycolytic function, cells were cultured as above without glucose and pyruvate. Then, glucose (10 mM), oligomycin (1 μM) and 2-deoxy-glucose (100 mM) were added sequentially. Oligomycin stops mitochondrial ATP synthesis and shifts the energy production pathway to glycolysis, with the subsequent increase in PPR revealing the cellular maximum glycolytic capacity. 2-deoxy-glucose, a glucose analog inhibits glycolysis by binding to hexokinase. To evaluate the β-oxidation of endogenous fatty acids we used the inhibitor of carnitine palmitoyltransferase, etomoxir (100 μM, Sigma), which prevents the formation of acyl carnitines, a step necessary for the transport of fatty acyl chains from the cytosol to the mitochondria. The QO_2_ was measured as above after the addition of etomoxir in infected and control cells. All experiments were performed three times independently.

### Oxidants Determination

Cells were incubated with *T. cruzi* and washed 2 h after, to remove free parasites. 24 h later cells were washed with RPMI and incubated with 100 μM (45 min at 37°C) 2′7′-dichloro-dihydro-fluorescein diacetate (DCFH-DA; Sigma); DCFH-DA oxidation by reactive oxygen species (ROS) was analyzed by flow cytometry.

### *T. cruzi* Infectivity Assays

Cardiomyocytes were treated with different concentrations (from 0.625 to 5 μM) of rapamycin (Sigma) for 24 h. Then the cells were washed, plated and infected with β-galactosidase expressing trypomastigotes at a ratio of 10 parasites to 1 cell. After 2 h of infection, non-internalized parasites were washed out. After 48 h, monolayers were washed and assays were developed using CPRG as a β-galactosidase substrate as previously described (Faral-Tello et al., 2014), and quantified by measuring the absorbance at 570 nm using an ELx800 Universal Microplate Reader (BioTek Instruments Inc., Winooski, VT, United States). Wells with no drug were considered as the 100% of parasite replication.

### Statistical Analysis

Statistical significance between two groups was estimated using the unpaired Student’s *t*-test. Differences in the bioenergetic profiles were calculated by XGe-96 software and GraphPad Prism software, using one-way ANOVA and Student’s *t*-test calculations. A *p*-value less than 0.05 was considered significant in all cases.

## Results

### Energy Metabolism and Protein Synthesis Related Genes Are Strongly Up-Regulated in Response to *T. cruzi* Infection

**FIGURE 1:**
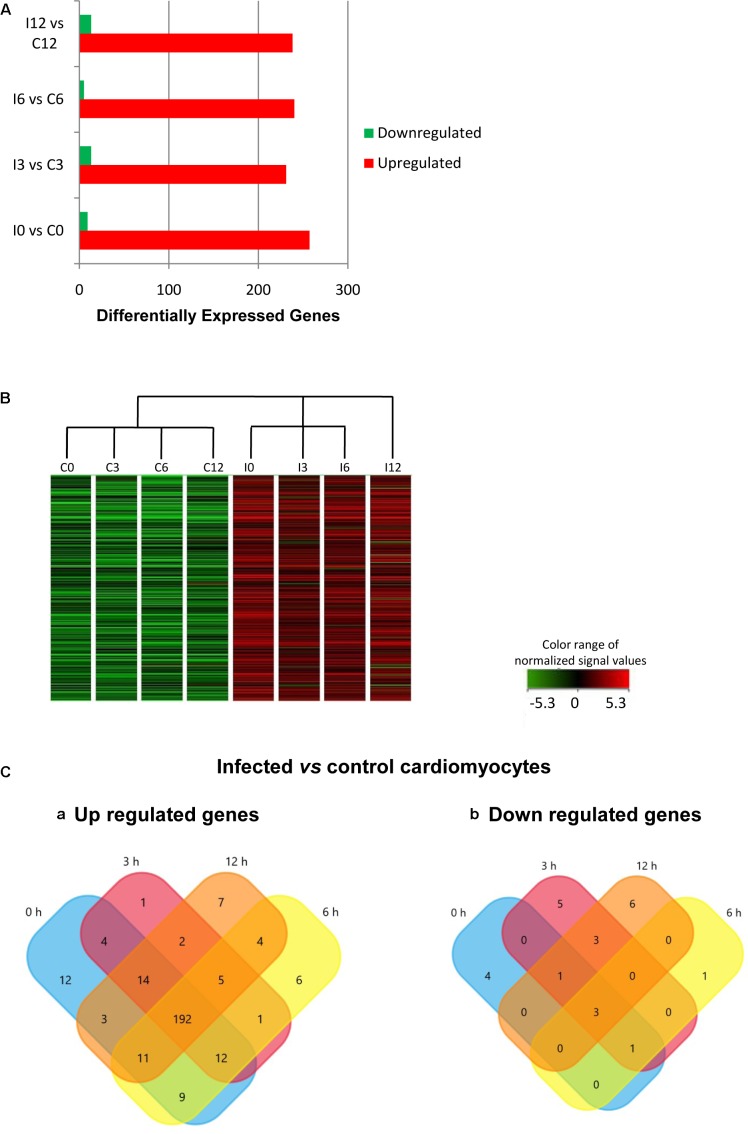
Differential gene expression in response to *Trypanosoma cruzi* infection. **(A)** Comparative expression at times 0, 3, 6, and 12 hpi (fold change ≥ 2; *p* ≤ 0.01). **(B)** Hierarchical clustering of the differentially expressed genes. **(C)** Venn diagrams comparing **(a)** up-regulated and **(b)** down-regulated genes from infected vs. control cardiomyocytes at the different times post infection.

**FIGURE 2:**
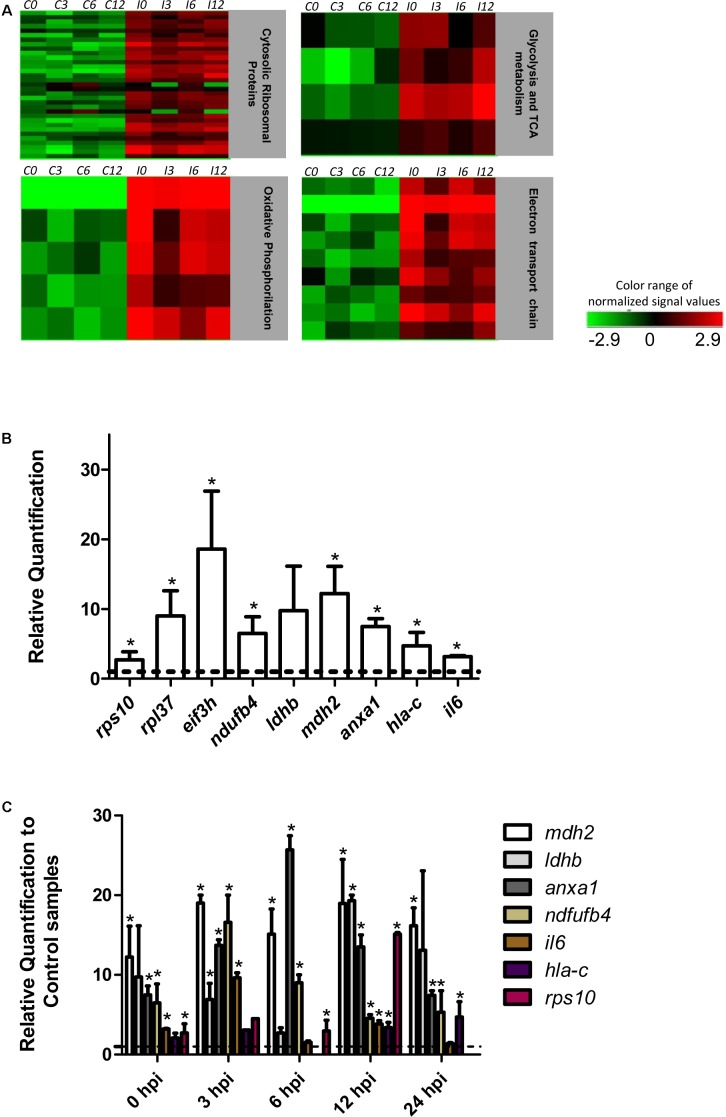
Pathway analysis and RT-qPCR. **(A)** Heat map of the most significantly altered pathways from the differentially expressed genes (*p* ≤ 0.01 Fc ≥ 2). C and I denotes control and infected cardiomyocytes respectively. **(B)** Quantification of selected genes by real-time PCR. The bars shows the relative fold changes of up-regulated genes *(rps10, rpl37, eif3h, ndufb4, ldhb, mdh2, anxa1, hla-c)* analyzed by qPCR at *t*_0_. Values are means of three biological replicates ± SEM. ^∗^*p* ≤ 0.05. The dotted line shows the value at which there is no difference in expression (1) between infected and control cells. **(C)** Kinetic expression analysis between 0–24 hpi in respect to each control by qPCR for selected genes. The dotted line shows the value at which there is no difference in expression (1) between infected and control cells. Values are means of two or three biological replicates ± SEM. ^∗^*p* ≤ 0.05.

The effect of *T. cruzi* infection on gene expression in human primary cardiomyocytes was investigated at 0 h (invasion phase), 3 h (intra-phagosomal amastigotes in the non-replicative phase), 6 h (release of amastigotes to host cytoplasm) and 12 h (cytosolic amastigotes, replicative phase) post-interaction. Cells incubated for similar number of hours without *T. cruzi* were used as controls. Genes showing at least a twofold change in their expression and a 99% probability of being differentially expressed (*p* ≤ 0.01) were considered (**Supplementary Table S2**). Our data showed that more than 290 genes were up-regulated in response to *T. cruzi* infection whereas less than 25 genes were down-regulated (**Figure 1A**). The hierarchical clustering of the differentially expressed genes clearly discriminated between control and infected cardiomyocytes (**Figure 1B**). More than 250 genes were up-regulated in expression in infected cardiomyocytes at 0 h post-interaction phase, and a majority of these remained up-regulated (**Figure 1Ca**) and only 3 remained down-regulated (**Figure 1Cb**) with progression of infection phase. Gene Ontology and pathway analysis showed important changes in the expression of genes related to protein synthesis and energy metabolism, mainly electron transport chain, oxidative phosphorylation and Krebs cycle, were significantly increased in expression in infected cardiomyocytes (**Table 1** and **Figure 2A**). Representative genes of each pathway were further confirmed by real time PCR (**Figure 2B**). We also did an expression kinetic analysis for some of the genes related to energy metabolism, (*mdh2*, *ldhb*, and *ndufb4*) finding that they were overexpressed between 0 and 24 hpi (**Figure 2C**).Together, the results presented in **Figure 2**, and **Table 1** indicate that cardiomyocytes respond to *T. cruzi* invasion with an immediate increase in the expression of genes related to mitochondrial oxidative metabolism, and this increase in oxidative metabolic gene response is maintained during parasite replication within the infected cardiomyocytes.

**Table 1:** Expression of energy metabolism associated genes.

Gene	Complex/location	0 h	3 h	6 h	12 h
ATP5E	Complex V	40.1	31.4	31.1	24.1
ATP6	Complex V	13.4	6.1	6.2	8.5
COX6c	Complex IV	5.4	3.4	6.1	7.0
NDUFB4	Complex I	9.2	3.2	4.8	7.7
NDUFB8	Complex I	4.8	4.2	3.7	3.9
CYTB	Complex III	3.9	5.2	1.8	3.2
UQCRFS1	Complex III	5.0	1.8	2.1	3.7
LDHB	Cytosolic	11.7	13.0	7.5	4.0
MDH2	Mitochondrial	7.9	10.2	6.5	10.7


**The values represent the Fold changes (Fc) of infected vs. control cells obtained with the Agilent microarray experiment (p ≤ 0.01 and Fc ≥ 2).**

### Increased Cardiomyocyte Mitochondrial Energy Metabolism as an Early Response to *T. cruzi* Infection

**FIGURE 3:**
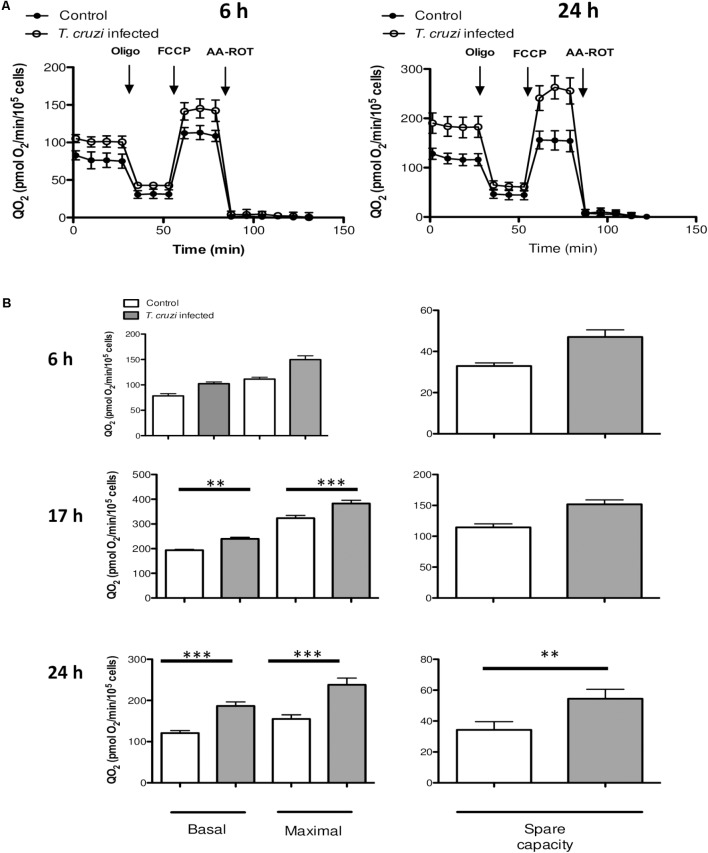
Mitochondrial bioenergetics. **(A)** QO_2_ were evaluated in control (filled circles) and infected (open circles) cells at 6 or 24 hpi following the addition of oligomycin (2 μM), FCCP (1 μM) and AA-ROT (1 μM each). QO_2_ is expressed as pmol O_2_ consumed/min/10^4^ cardiomyocytes and are the mean of three independent determinations. **(B)** Mitochondrial bioenergetics parameters were calculated after subtraction of non-mitochondrial respiration (QO_2_ after AA-ROT addition) to all data. Basal respiration (no drug added), maximal respiration (QO_2_ after FCCP addition) and spare respiratory capacity (calculated as the ratio between QO_2FCCP_ and QO_2BASAL_) at the different time points analyzed (6, 17, and 24 hpi). Each data point represent the mean ± SEM *n* = 10. ^∗∗^*p* < 0.01, ^∗∗∗^*p* < 0.001.

To determine how the changes in gene expression impact the metabolic profile of infected cardiomyocytes, we evaluated oxidative energy metabolism by using the extracellular flux analyzer technology. Infected and control cardiomyocytes were cultured in un-buffered DMEM in the presence of glucose, pyruvate and glutamine, and the QO_2_ was measured after the sequential addition of oligomycin (inhibitor of ATP synthase, complex V), FCCP (uncoupling agent) and a mix of antimycin A (AA, complex III inhibitor) and rotenone (Rot, complex I inhibitor). As indicated in material and methods the basal respiration can be derived by subtracting to the initial OCR the non-mitochondrial respiration. The maximal respiratory capacity is derived by subtracting non-mitochondrial respiration from the FCCP rate and the spare capacity is calculated by subtracting basal respiration from maximal respiratory capacity. No significant differences were observed in QO_2_ between normal vs. infected cells at 6 hpi, whereas by 24 hpi, cardiomyocytes exhibited a substantial increase in QO_2_ of respiration (**Figure 3**). As early as 17 hpi, the basal and maximal QO_2_ as well as the spare respiration capacity were significantly increased in infected cells as compared to that noted in matched, control cardiomyocytes (**Figure 3B**). In order to evaluate if these changes corresponded to a general response to *T. cruzi* infection, we performed the same assay in previously studied HeLa cells (Chiribao et al., 2014) at 24 hpi, and no significant differences were observed in QO2 values (**Supplementary Figure S1**).

**FIGURE 4:**
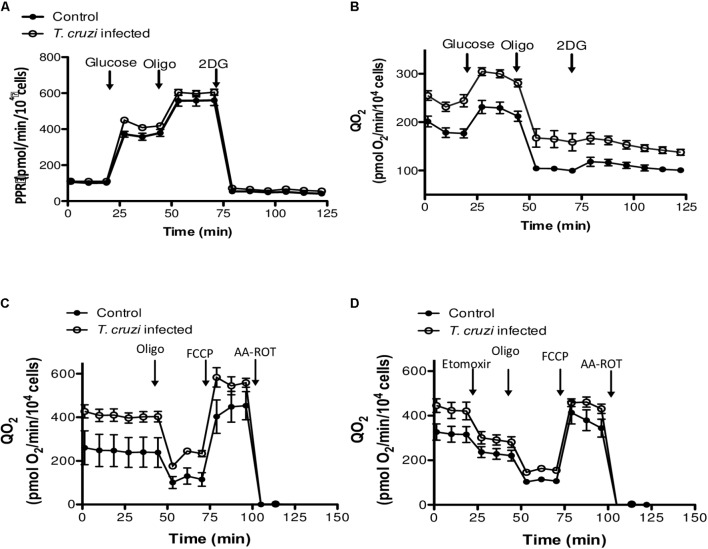
Evaluation of glucose and fatty acid metabolism. **(A)** Kinetic of the PPR response of control and *T. cruzi* infected cardiomyocytes (24 hpi) to glucose (10 mM), oligomycin (2 μM) and 2-DG (100 mM). **(B)** Cardiomyocyte QO_2_ response of control and *T. cruzi* infected cardiomyocytes (24 hpi) to glucose (10 mM), oligomycin (2 μM) and 2-DG (100 mM). **(C,D)** Kinetic of QO_2_ response in the absence (E) or presence (F) of the CPT1 inhibitor etomoxir (100 μM), oligomycin (2 μM), FCCP (1 μM), and AA-ROT (1 μM each) in control and *T. cruzi* infected cardiomyocytes at 24 hpi. Culture media was the Human Cardiomyocytes Primary Cell Culture (Celprogen).

We then studied the contribution of glycolysis to the enhanced respiratory activity, through measurements of QO_2_ and PPR when glucose was added as the only exogenous energy carbon source, in the presence or absence of 2-deoxy glucose (2DG). No significant differences in the glycolytic capacity of control and *T. cruzi* infected cardiomyocytes were observed (**Figures 4A,B**). Finally, to evaluate the oxidation of endogenous fatty acids as energy source, we compared mitochondrial QO_2_ in the presence or absence of etomoxir and, as for the glycolytic pathway, no differences were observed in the capacity of fatty acid oxidation (**Figures 4C,D**). Together, these results suggest that the cardiomyocytes respire more to support the electron transport chain linked oxidative phosphorylation in response to infection by *T. cruzi.*

### *T. cruzi* Infection Induces Mitochondrial Biogenesis in Human Cardiomyocytes

**FIGURE 5:**
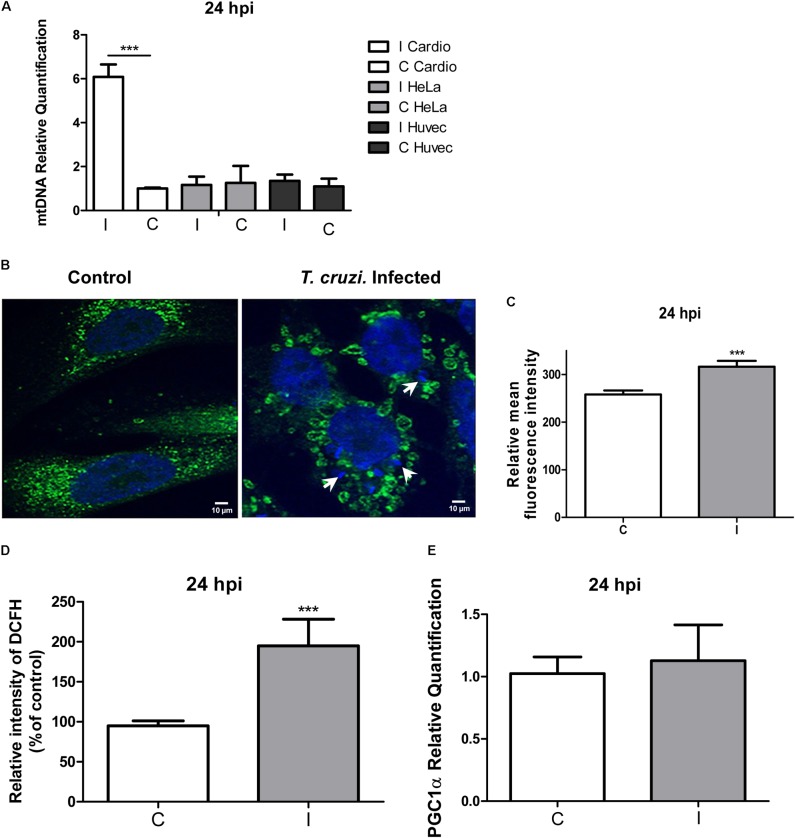
Mitochondrial biogenesis and ROS evaluation. **(A)** Quantification of the mtDNA relative to the nuclear DNA in infected cardiomyocytes, HeLa and primary Huvec cells versus the respective controls at 24 hpi determined by qPCR. Values are means of three biological replicates ± SEM. ^∗∗∗^*p* ≤ 0.001. **(B)** Confocal microscopy using anti-cytochrome c antibody (dilution 1/100) and DAPI for DNA staining after 24 hpi. White arrows denote the presence of intracellular amastigotes. **(C)** Measure of the means ± SD of the fluorescence intensity of infected and control cells. ^∗∗∗^*p* ≤ 0.001. **(D)** The production of ROS at 24 hpi was monitored by DCF fluorescence. Results are expressed graphically as percentages of the fluorescence intensity of the control cells. Values are means ± SEM of the results from three independent experiments performed in triplicate (C = uninfected and I = infected cells; ^∗∗∗^*p* < 0.002). **(E)** Relative quantification of *pgc-1*α by qPCR, showing the relative fold change of *T. cruzi* infected cells (I) vs. control cardiomyocytes (C) at 24 hpi. Values represents the mean of three independent biological replicates.

We next determined if the increase in the QO_2_ in infected cardiomyocytes could be related to mitochondrial biogenesis. For this purpose we compared a specific DNA region of the mitochondrial genome relative to a nuclear gene (see section “Materials and Methods”), and we found a sixfold increase in mitochondrial DNA in infected cardiomyocytes (**Figure 5A**). Mitochondrial biogenesis was further explored by immunofluorescence microscopy using anti-cytochrome C antibody, and the results clearly evidenced an increase in the mitochondrial content (**Figures 5B,C**). Interestingly, we observed the presence of ring-like donut mitochondria in infected cardiomyocytes, which could be associated to a mild increase in mitochondrial oxidant generation (Liu and Hajnoczky, 2011; Ahmad et al., 2013). To elucidate whether cellular oxidants were increased in infected cardiomyocytes, we used the DCFH-DA probe (detects intracellular ROS), and we found a twofold increase in the relative intensity of DCF fluorescence (**Figure 5D**). Finally, since PGC-1α is described as a regulator of mitochondrial biogenesis (Fernandez-Marcos and Auwerx, 2011), we compared its expression in infected and non-infected cardiomyocytes, and no significant differences were found (**Figure 5E**). These results suggest that cardiomyocytes infected by *T. cruzi* increase the PGC-1α independent mitochondrial biogenesis to support the increased oxidative respiration and consequent increase in ROS production.

When HeLa cells were analyzed, no changes at the mtDNA level were found (**Figure 5A**). We also evaluated infected HUVEC cells, in order to include another primary human cell in the assay, and no changes in mtDNA content were observed (**Figure 5**). Taken together these results indicate that mitochondrial biogenesis probably constitutes a cardiomyocyte specific response to *T. cruzi* infection.

### Activation of the AKT/mTORC1 Signaling Pathway in Infected Cardiomyocytes

**FIGURE 6:**
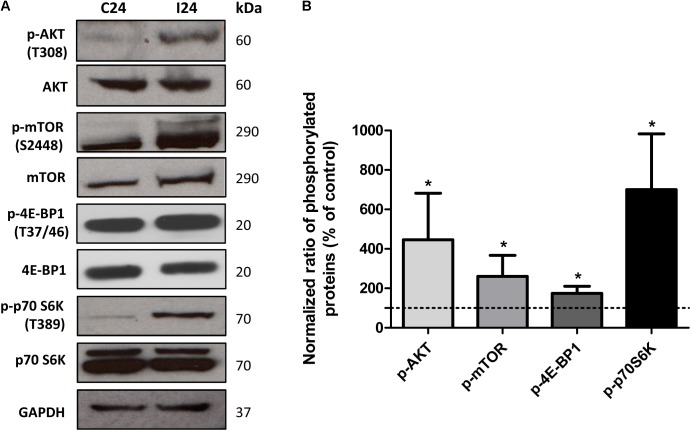
Analysis of AKT/mTORC1 pathway. **(A)** Western blot analysis of phosphorylated and non-phosphorylated AKT, mTOR, 4EBP1, p70S6k in control and *T. cruzi* infected cardiomyocytes at 24 hpi. GAPDH was used as loading control. **(B)** Densitometry analysis of western blots. Values represents the mean ± standard deviation of three or two independent biological replicates (^∗^*p* < 0.05).

**FIGURE 7:**
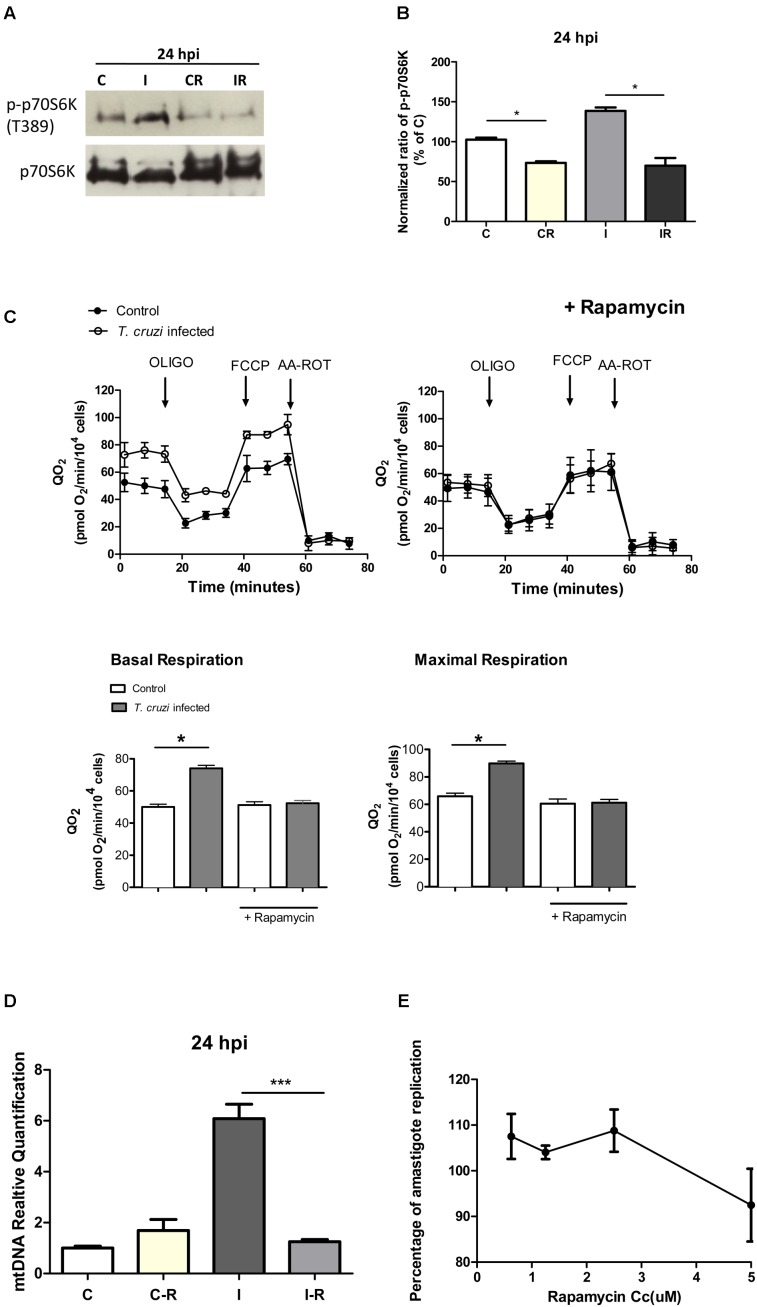
Effect of rapamycin. **(A)** Western blot analysis of phosphorylated and non-phosphorylated p70S6k in control and infected cardiomyocytes at 24 hpi (C, I), vs. the same cells treated with rapamycin for 24 h before the infection (CR, IR). **(B)** Densitometric analysis of two independent experiments showing p-p70S6k protein expression normalized against the non-phosphorylated protein in control cells (^∗^*p* < 0.05). **(C)** Kinetic of QO_2_ response to oligomycin (2.0 μM), FCCP (1.0 μM) and AA-ROT (1.0 μM each) in control and *T. cruzi* infected cardiomyocytes cells 24 hpi with and without Rapamycin treatment (2.5 μM for 24 h before infection). Arrows indicates the injection of the different additions. Basal respiration (no drug added) and maximal respiration (QO_2_ after FCCP addition) with and without the rapamycin treatment were calculated. Each data point represent the mean ± SEM *n* = 5. ^∗^*p* < 0.05. **(D)** Relative fold changes of the mtDNA gene relative to the nuclear gene beta actin in infected cardiomyocytes (I), infected cardiomyocyte treated with rapamycin (2.5 μM) 24 h before infection (IR), and controls treated with rapamycin (2.5 μM) (CR) versus control cells at 24 hpi (C). Values are the mean of three biological replicates ± SEM (^∗∗∗^*p* < 0.0002). **(E)** Percentage of amastigote replication and trypomastigote infection in comparison with an untreated control (100%). Cardiomyocytes were treated with different rapamycin concentrations for 24 h, washed and infected with *T. cruzi.* Each point of the curve represents the mean ± standard deviation of three independent experiments.

In addition to an increase in mitochondrial energy metabolism, several of the genes related to protein synthesis were also up-regulated in cardiomyocytes during early infection phase (**Figure 2** and **Supplementary Table S3**). This observation led us to hypothesize that mTORC1, an AKT dependent signaling cascade that coordinates both mitochondrial activity and protein synthesis (Laplante and Sabatini, 2009), might be activated in infected cardiomyocytes. We therefore evaluated the activation of mTORC1 pathway by studying the phosphorylation state of different members of this cascade. Our data showed that at 24 hpi, AKT/mTORC1 cascade was activated, as evidenced by the higher levels of phospho-AKT, phospho-mTOR, phospho-4EBP1, and phospho-p70S6 kinase in infected (vs. control) cardiomyocytes (**Figure 6**). To test if mTORC1 activation was involved in mitochondrial bioenergetics increase, we treated the infected cardiomyocytes with rapamycin (specific inhibitor of mTORC1). We first confirmed that rapamycin inhibits mTORC1 phosphorylation in our experimental conditions (**Figures 7A,B**). We then measured the oxygen consumption rate, and found that treatment with rapamycin inhibited the *T. cruzi* induced increase in the maximal respiration and spare reserve capacity in infected cardiomyocytes, and infected cells exhibited similar levels of respiration as was noted in controls (**Figure 7C**). Moreover, the relative increase in mtDNA content observed in infected (vs. normal control) cells was subsided when infected cardiomyocytes were treated with rapamycin (**Figure 7D**).

Finally, we analyzed whether treatment with rapamycin affects the infection, and we did not find significant differences in the burden of intracellular amastigotes in the presence of the drug (**Figure 7E**). Rapamycin treatment did not affect the viability of the cells (**Supplementary Figure S2A**) and did not inhibit mTORC2 (**Supplementary Figure S2B**) in these conditions.

## Discussion

Chronic cardiomyopathy is the most severe manifestation of Chagas disease (Rassi et al., 2017). In the heart *T. cruzi* is able to infect a wide variety of cells including endothelial cells, smooth muscle cells, fibroblasts, and cardiomyocytes (Mukherjee et al., 2004). It has long been demonstrated through histological analysis of biopsies, that morphological changes in cardiomyocytes such as myofiber hypertrophy, had a good correlation with the clinical manifestation and severity of heart disease (Higuchi et al., 1987; Higuchi, 1995). In the present study we found that *T. cruzi* remodeled the global pattern of gene expression in human cardiomyocytes, by up-regulating hundreds of genes immediately after infection, being mitochondrial energy metabolism and protein synthesis the most up-regulated pathways. When similar experiments were performed on HeLa cells (Chiribao et al., 2014), a completely different landscape was found: the most up-regulated pathways were related to immune/inflammatory responses, indicating that *T. cruzi* is able to remodel host gene expression through cell type-specific programs.

We found here an increase in basal and maximal respiration, as well as in spare respiratory capacity, which is accompanied by mitochondrial biogenesis. Remarkably, our transcriptomics results significant differ from previous studies in murine cardiomyocytes. Manque et al. (2011) found that the most up-regulated pathways in the early infection were related to a inflammatory response, whereas mitochondrial energetic related genes did not change significantly. Also by using murine cardiomyocytes a decrease in oxidative phosphorylation was reported (Vyatkina et al., 2004; Estrada et al., 2018). However, our results are in agreement with gene profiling studies on human heart biopsies. Indeed, comparison between patients whit CCC and DCM showed that CCC and not DCM, is characterized by an up-regulation of genes involved in oxidative phosphorylation and proton transfer chain, and both diseases exhibit up-regulation of protein synthesis related genes (Cunha-Neto et al., 2005). Furthermore, a similar analysis comparing myocardial tissues from HCM vs. healthy patients showed that the most altered pathways were protein synthesis and oxidative phosphorylation (Yang et al., 2015). Remarkably, the two HCM molecular marker genes matrix metalloproteinase 1 (*mmp1*) (Miura et al., 2003), and Atrial Natriuretic Peptide (*npr1*) (Zoccali et al., 2001) were up-regulated in response to infection (see **Supplementary Table S2**). These results suggest that the model used in this work may be adequate for studying molecular mechanisms of pathogenesis, and that cardiomyocyte gene expression remodeling in response to infection contributes to molecular basis of Chagas heart disease.

The AKT dependent mTORC1 pathway regulates mitochondrial activity and biogenesis, as well as protein synthesis (Zoncu et al., 2011; Roux and Topisirovic, 2012; Morita et al., 2013, 2015; Nandagopal and Roux, 2015). Since most of the ribosomal proteins and translation factors coding genes were induced upon infection, we hypothesized that mTORC1 was at the basis of these changes, and we clearly showed its activation, whereas mTORC2 remained unchanged in both conditions. The direct implication of mTORC1 was further demonstrated by the use of rapamycin: the presence of the drug abolished the differences between infected and non-infected cells, both at the metabolic level and mtDNA content. Changes in host mTOR related pathways in response to *T. cruzi* infection have been already described in other cell lines such as HeLa (Caradonna et al., 2013) and macrophages (Rojas Marquez et al., 2018). However, the consequences of this activation are different depending on the cell type, which reinforces the concept of the infection plasticity of this parasite, which is not only able to infect almost any kind of cells but also, by inducing similar pathways, each cell or tissue has specific signal transduction hallmarks. In fact, we further confirmed that neither HeLa cells nor primary HUVEC cells showed a mitochondrial biogenesis phenotype. As mentioned, mitochondrial biogenesis was found to be directly related to mTOR activation: when cells were incubated with rapamycin the mtDNA content in infected cells vs. controls did not differ, and correspond to a *pgc-1*α independent mechanism of mitochondria biogenesis, previously described (Morita et al., 2013). It is noteworthy that parasite burden did not change in presence of rapamycin, which suggests that increase in oxidative respiration and subsequent increase in ROS production do not seem to constitute a host defense mechanism, but a side effect that damages the cardiomyocytes, leading to a pathological phenotype (Gupta et al., 2009; Roux and Topisirovic, 2012). Interestingly, reversion of the pathological phenotype by rapamycin draws our attention about host-targeted therapies in Chagas disease, where most of the efforts have been oriented to the parasitological cure. However, since its most severe manifestation is heart damage, which take years to manifest clinical signs, the understanding of molecular mechanisms of pathogenesis of CCC may allow to identify potentially new targets in the hosts, for developing new therapeutic strategies of combined treatment focused toward both parasitological and clinical cure. Similar strategies are already being assayed in other intracellular and persistent pathogens, as for example *Mycobacterium tuberculosis* (Jayachandran et al., 2012; Tomioka, 2014).

In conclusion, a key finding of this work is the ability of *T. cruzi* to induce an mTORC1 mediated increase in respiration and mitochondrial content, immediately after infection of human cardiomyocytes. This altered phenotype resembles the main differences found between HCM vs. normal cardiac tissues, and CCC vs. DCM, which lead us to propose that these changes are at the basis of the molecular mechanism of pathogenesis in CCC. The finding that the drug rapamycin can revert this phenotype opens a promising perspective in the treatment of Chagas disease: the use of combined and complementary parasite and host targeted therapies.

## Author Contributions

ML: data collection, analysis and interpretation, and wrote the article. PF-T: cell culture, infection experiments, and interpretation. NG: design of the work and critical revision of the article. RR: data interpretation and critical revision of the article. LP: seahorse experiments, analysis, interpretation, and wrote the article. CR: conception and design of the work, data interpretation, and wrote the article.

## Conflict of Interest Statement

The authors declare that the research was conducted in the absence of any commercial or financial relationships that could be construed as a potential conflict of interest.

## Supplementary Material

FIGURE S1 QO_2_ in HeLa cells. Kinetic of QO_2_ response to oligomycin (2 μM), FCCP (1 μM) and AA-ROT (1 μM each) in control and *Trypanosoma cruzi* infected HeLa cells 24 hpi. Arrows indicates the injection of the different additions. No statistical differences were observed in any mitochondrial bioenergetics parameters between both conditions.

FIGURE S2 Evaluation of cell viability and mTORC2 after Rapamycin treatment. **(A)** Resazurin (Sigma) reduction was used to evaluate the viability of the cells treated with rapamycin at different concentrations for 24 h. The viability of the cells without rapamycin was considered as 100%. **(B)** mTORC1 and mTORC2 evaluation in cardiomyocytes treated with rapamycin (2.5 μM) for 24 h, washed and infected with *T. cruzi*. The proteins were collected 24 hpi. The relative p-AKT (ser 473) expression was normalized against total AKT for mTORC2 evaluation. The same membrane was stripped and evaluated for mTORC1 with the p-p70S6k antibody normalized against the non-phosphorylated p70S6k. Densitometry analysis of mTORC2 western blots. Values represents the mean ± standard deviation of two independent biological replicates.

TABLE S1 Primer sequences and expected product length.

TABLE S2 Microarray differentially expressed genes.

TABLE S3 Differentially expressed genes related to Ribosomal proteins and Translation factors (Fc ≥ 2, *p* ≤ 0.01).
