# Supplementary figures and images for: Early Trypanosoma cruzi Infection Triggers mTORC1-Mediated Respiration Increase and Mitochondrial Biogenesis in Human Primary Cardiomyocytes
Source: Front Microbiol. 2018 Aug 16;9:1889. doi: 10.3389/fmicb.2018.01889 (PMC6106620; doi:10.3389/fmicb.2018.01889)

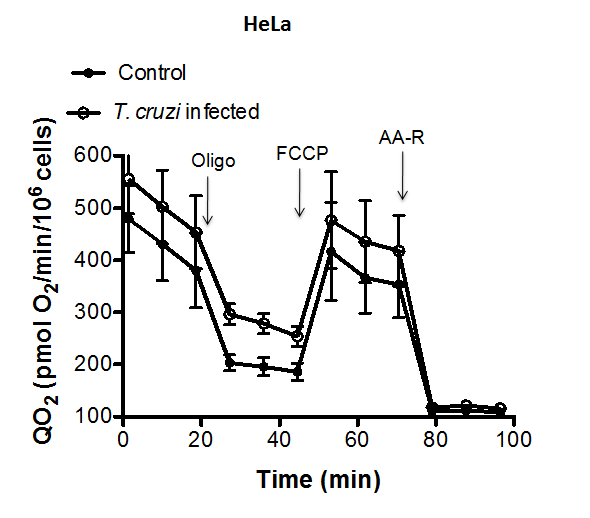

Supplement: FIGURE S1 — QO2 in HeLa cells. Kinetic of QO2 response to oligomycin (2 μM), FCCP (1 μM) and AA-ROT (1 μM each) in control and Trypanosoma cruzi infected HeLa cells 24 hpi. Arrows indicates the injection of the different additions. No statistical differences were observed in any mitochondrial bioenergetics parameters between both conditions. [file Image_1.tif]

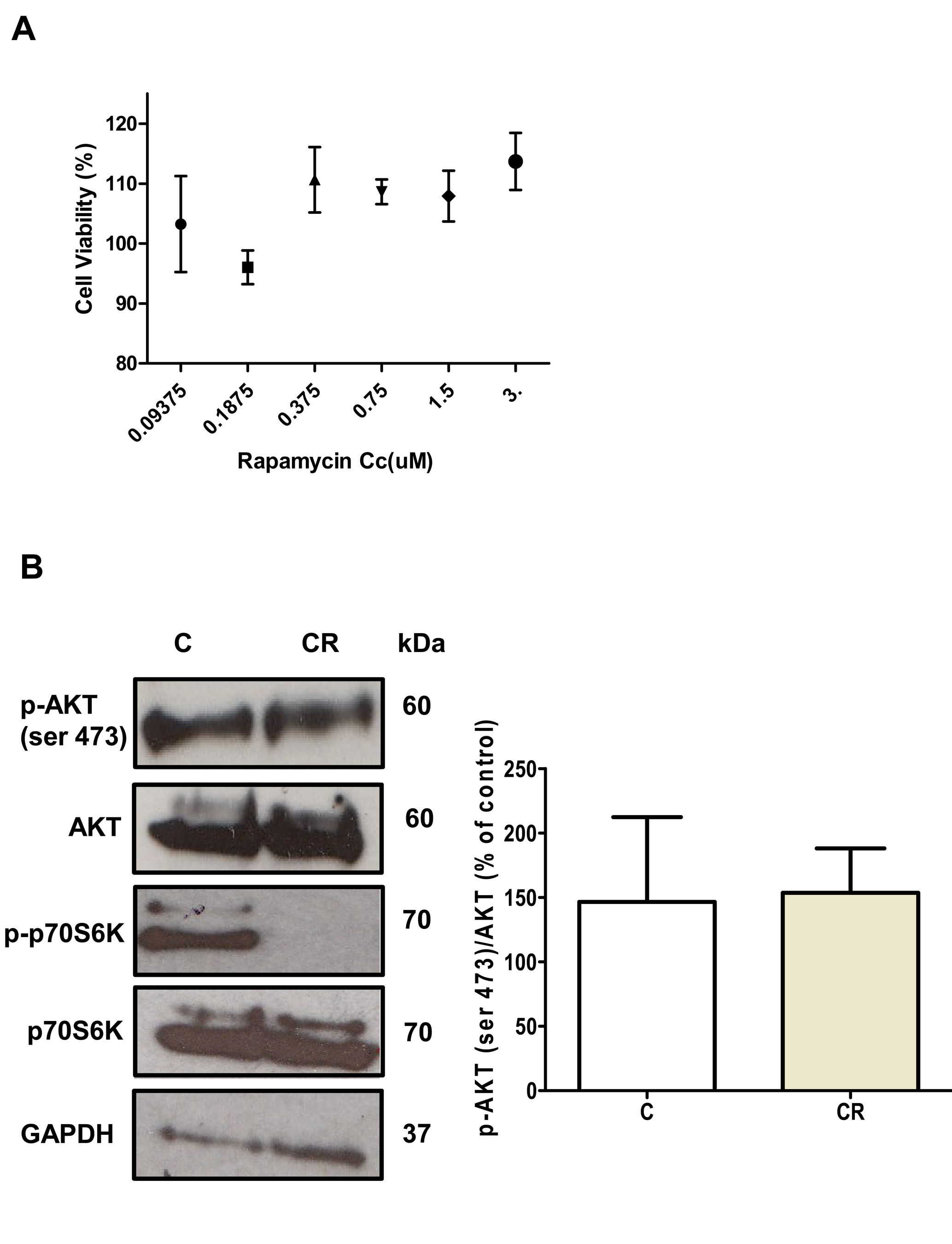

Supplement: FIGURE S2 — Evaluation of cell viability and mTORC2 after Rapamycin treatment. (A) Resazurin (Sigma) reduction was used to evaluate the viability of the cells treated with rapamycin at different concentrations for 24 h. The viability of the cells without rapamycin was considered as 100%. (B) mTORC1 and mTORC2 evaluation in cardiomyocytes treated with rapamycin (2.5 μM) for 24 h, washed and infected with T. cruzi. The proteins were collected 24 hpi. The relative p-AKT (ser 473) expression was normalized against total AKT for mTORC2 evaluation. The same membrane was stripped and evaluated for mTORC1 with the p-p70S6k antibody normalized against the non-phosphorylated p70S6k. Densitometry analysis of mTORC2 western blots. Values represents the mean ± standard deviation of two independent biological replicates. [file Image_2.tif]
