# Supplementary material for: Early Trypanosoma cruzi Infection Triggers mTORC1-Mediated Respiration Increase and Mitochondrial Biogenesis in Human Primary Cardiomyocytes
Source: Front Microbiol. 2018 Aug 16;9:1889. doi: 10.3389/fmicb.2018.01889 (PMC6106620; doi:10.3389/fmicb.2018.01889)
Supplement: TABLE S1 — Primer sequences and expected product length. [file Table_1.PDF]

Supplementary Table 1

Gene	Forward	Reverse	pb
ldhb	GTCTTCTCCGCACGACTGTT	CAGCCAGAGACTTTCCCAGAA	195
mdh2	ATGATATCGCGCACACACCC	GGGATGGTGGAATTAACCGGA	280
ndufb4	GCCATAAGAGCCCAGCTGAA	TCTTTCCTATCCCTCTCAGTTTTGA	227
anxa1	GCAAGAAGGTAGAGATAAAGACACT	TGACGCTGTGCATTGTTTCG	267
eif3h	CTTGGAAAGATGGCGTCCCG	AATACCACAAGGCCATCTATCTGC	149
rpl37	TGTATACCGCAGATTCAGGC	AAACCAGAACATTTATTGCATGAC	127
rps10	CGAGACTCACAAGAGGGGAA	ACTGGAATTCGGTTGCTGAC	123
pgc-1α	GACCCTCCTCACACCAAACCCACA	GGGGTCATTTGGTGACTCTGGGGTC	156
hla-c	ATCGTTGCTGGCCTGGCTGTCCT	TCATCAGAGCCCTGGGCACTGTT	146
$mt_{7121-7284}$	CAAACCTACGCCAAAATCCA	GAAATGAATGAGCCTACAGA	164
β-actin	TCACCCACACTGTGCCCATCTACGA	CAGCGGAACCGCTCATTGCCAATGG	295
