# Supplementary material for: Early Trypanosoma cruzi Infection Triggers mTORC1-Mediated Respiration Increase and Mitochondrial Biogenesis in Human Primary Cardiomyocytes
Source: Front Microbiol. 2018 Aug 16;9:1889. doi: 10.3389/fmicb.2018.01889 (PMC6106620; doi:10.3389/fmicb.2018.01889)
Supplement: TABLE S3 — Differentially expressed genes related to Ribosomal proteins and Translation factors (Fc ≥ 2, p ≤ 0.01). [file Table_3.PDF]

Supplementary Table 3

Ribosomal Proteins				
**Gene**	**0h**	**3h**	**6h**	**12h**
**RPS10**	28.2	22.4	15.4	17.6
**RPS15A**	22.2	21.8	11.8	15.4
**RPS26**	20.7	10.9	21.9	18.4
**RPS17**	20.4	7.3	26.9	17.4
**RPS13**	17.0	13.4	12.7	15.9
**RPS7P5**	11.7	3.1	11.5	14.2
**RPS29**	11.0	6.4	4.0	7.8
**RPS10**	10.9	8.0	14.8	19.0
**RPS2**	6.9	4.6	4.0	4.4
**RPS7**	6.7	7.0	4.4	8.6
**RPS6**	6.4	2.5	2.2	4.9
**RPS23**	5.1	2.1	3.1	4.0
**RPS28**	4.4	3.6	9.1	5.3
**RPS10**	4.1	3.2	6.1	4.8
**RPS2P32**	3.9	3.3	4.1	4.1
**RPS10**	3.6	7.6	2.8	7.2
**RPL14**	15.6	5.3	6.0	8.3
**RPL37**	15.4	6.8	13.7	14.4
**RPL7**	13.4	5.1	6.1	8.1
**RPL17**	12.3	16.5	8.8	14.3
**RPL39**	8.5	11.0	6.6	7.7
**RPL35**	7.7	9.3	8.1	11.3
**RPL29**	6.9	11.4	4.1	16.7
**RPL36**	6.9	5.9	7.2	9.2
**RPL18**	5.8	2.4	1.8	2.5
**RPL29**	5.5	4.7	5.7	9.4
**RPL36A**	5.2	2.6	3.6	2.8
**RPL39**	5.0	5.8	4.1	3.4
**RPL13AP3**	4.9	1.9	2.2	2.0
**RPL41**	4.9	3.9	4.0	4.5
**RPL32**	4.9	4.8	5.4	7.0
**MRPL43**	3.8	2.2	5.1	3.3
**RPL14**	1.9	2.3	1.8	3.2
**Translation factors**				
**EIF3H**	10.2	13.5	7.7	8.9
**EIF3D**	8.8	10.9	7.2	8.2
**EIF3M**	2.8	3.7	3.0	3.6
**PABPC1**	26.0	32.1	12.9	28.9
